# Post-Traumatic Osteoarthritis and Functional Outcomes After Volar Plating vs. Casting of Unstable Distal Radius Fractures: A Minimum 2-Year Follow-Up of the VOLCON Randomized Controlled Trial

**DOI:** 10.3390/jcm14113766

**Published:** 2025-05-28

**Authors:** Daniel Wæver, Rikke Thorninger, Karen Larsen Romme, Michael Tjørnild, Jan Duedal Rölfing

**Affiliations:** 1 Department of Orthopaedics, Regional Hospital Randers, Skovlyvej 15, Randers NØ, 8930 Randers, Denmark; karrom@rm.dk (K.L.R.); michael.tjornild@rm.dk (M.T.); 2 Department of Orthopaedics, Aarhus University Hospital, Aarhus N, 8200 Aarhus, Denmark; rikkthor@rm.dk (R.T.); jan.rolfing@rm.dk (J.D.R.); 3 Department of Clinical Medicine, HEALTH, Aarhus University, Aarhus C, 8000 Aarhus, Denmark

**Keywords:** distal radius fractures, post-traumatic osteoarthritis, randomized controlled trial, non-operative treatment

## Abstract

**Background/Objectives**: Distal radius fractures (DRFs) are among the most common fractures in the elderly, with increasing incidence due to population aging. Recent evidence questions the benefits of operative treatment, particularly in elderly patients. The present study aimed to assess post-traumatic osteoarthritis (OA) and patient-reported outcome measures (PROMs) after a minimum of two years of follow-up of the previously published VOLCON randomized controlled trial (RCT), which compared operative and non-operative treatments of unstable DRFs in patients aged ≥ 65 years. **Methods**: This study presents a minimum two-year follow-up of a single-center, assessor-blinded RCT. A total of 100 patients with unstable DRFs were randomized to either operative treatment with volar locking plating or non-operative treatment with cast immobilization. The primary outcome was post-traumatic OA, assessed using the Knirk and Jupiter classification. Secondary outcomes included PROMs (Quick Disabilities of the Arm, Shoulder, and Hand (Quick-DASH)) and Patient-Rated Wrist/Hand Evaluation (PRWHE), complications, pain, grip strength, and range of motion (ROM). Statistical analyses were performed using two-way ANOVA. **Results**: After a median follow-up of 3.0 years, 60 patients (28 non-operative and 32 operative) were available for analysis. There was no significant difference in OA between the groups (*p* = 0.57). PROMs (Quick-DASH, PRWHE), pain, grip strength, and ROM were time-dependent (*p* < 0.001) but not treatment-dependent. Complications were more frequent in the operative group, including hardware-related issues requiring reoperation. **Conclusions**: At a minimum of two years of follow-up, no correlation was found between treatment choice and post-traumatic OA. Functional outcomes were similar between groups, suggesting that non-operative treatment remains a viable option for elderly patients with unstable DRFs.

## 1. Introduction

Distal radius fractures (DRFs) are among the most common fractures in the elderly with incidence rates of 190–200 per 100,000 person-years [1,2]. These incidence rates are expected to increase with the projected aging population [3].

In Denmark, operative treatment was recommended by the National Clinical Guidelines (NCG) (2017) for unstable DRFs when the following radiological criteria were fulfilled after attempted closed reduction in the emergency department (ED) [4,5]: >10° dorsal tilt of the radius, >2 mm articular step-off, >3 mm ulnar variance, incongruence of the distal radioulnar joint, and substantial dorsal comminution indicating gross instability.

For the past few decades, open reduction and internal fixation (ORIF) with volar locking plates has becoming increasingly popular [6,7] and is now the operative treatment of choice. Studies have shown that ORIF improves radiological alignment and restores the distal radius closer to normal anatomy [8,9]. However, recent high-quality studies have questioned the benefits of operative treatment for DRFs in the elderly, and evidence supporting non-operative treatment is mounting [10,11,12,13,14].

Three studies have had an observation period of more than one year of elderly patients with unstable DRFs randomized to either operative or non-operative treatment. However, these studies have shown conflicting results [15,16,17].

The present study is a minimum 2-year follow-up of a previously published randomized controlled trial (VOLCON RCT) [14] comparing operative and non-operative treatments of unstable DRF in patients ≥ 65 years. Given the poorer radiological parameters in terms of angulation and shortening in the non-operatively treated group at 5-week follow-up, it seemed logical to reinvite the VOLCON patients and assess their post-traumatic osteoarthritic changes and patient-related outcome measures (PROMs) after a longer follow-up period of more than one year.

In the present study, we wanted to assess the explorative outcomes of the original RCT. Therefore, we assessed PROMs as well as radiological osteoarthritis (OA) changes after non-operative and operatively treated patients after a minimum of two years of follow-up.

## 2. Materials and Methods

This study describes the minimum 2-year follow-up on an already published prospective, single-center, assessor-blinded, randomized, controlled superiority trial comparing non-operative versus operative treatments of unstable DRFs in patients ≥ 65 years [14]. A study protocol was also published [18]. The primary study took place between November 2019 and March 2022.

Interventions and randomization

Patients with DRFs aged 65 years and above admitted to the ED at Randers Regional Hospital, Denmark, were screened for eligibility. Exclusion criteria were high-energy fractures, open fractures, concomitant injuries, previous ipsilateral DRFs, and the inability to give written consent for participation in the study.

DRFs were diagnosed on radiographs of the wrist (posterior–anterior and lateral projections), and closed reduction was performed with a hematoma block by the attending physician in the ED. A maximum of two attempts at closed reduction were allowed to obtain an acceptable reduction in the fracture. If the NCG criteria [4] for operation were fulfilled, the patient was randomized to either operative or non-operative treatment.

According to sample size calculations from the primary study, 100 patients were included. Thus, patients were blindly randomized by picking one of 100 identical sealed envelopes, each containing a note stating “operative” or “non-operative” written. The envelopes were non-transparent, and the concealment of allocation was therefore effective.

Patients allocated to operation were treated with ORIF with volar locking plate fixation (AcuLoc, Acumed, Hillsboro, OR, USA or VariAx, Stryker, Kalamazoo, MI, USA). All patients were operated on using a standard Henry approach for the distal radius. The repair of the pronator quadratus was performed when possible. Patients were operated under regional or general anesthesia. Post-operatively, the wrist was immobilized with a dorsal plaster cast for 2 weeks, followed by 3 weeks of immobilization with a removable wrist orthosis. A single session of hand therapy instruction took place.

Patients allocated to the non-operative treatment were immobilized with a dorsal plaster cast for 5 weeks. A single session of hand therapy instruction took place after cast removal. No radiographs were performed before the 5-week follow-up.

Outcomes

Primary and secondary outcomes were assessed for the primary study at day 0, 2 weeks, 5 weeks, 6 months, and 12 months after injury. For the present study, patients were followed-up at minimum 2 years after the injury. Patients were contacted by telephone and invited to participate. At the 2-year follow-up, the observers DW and KLR were blinded as all measurements were performed with the wrist covered by a glove to mask potential surgical scars.

### Explorative Outcomes of the Original RCT

The degree of post-traumatic OA in the radiocarpal and distal radioulnar joints was assessed by an orthopedic trauma specialist according to the Knirk and Jupiter classification [19] on a scale from 0 to 3 (0: none, 1: slight joint-space narrowing, 2: marked joint-space narrowing and osteophyte formation, and 3: bone-on-bone osteophyte formation and cyst formation) on standard radiographs of the wrist (posterior–anterior and lateral projections).

PROMs included a Danish version of the Quick Disabilities of the Arm, Shoulder, and Hand (Quick-DASH). A difference of 16–20 points in Quick-DASH was considered the minimum clinically important difference (MCID) [20,21,22]. The validated Danish version of the Patient-Rated Wrist/Hand Evaluation (PRWHE) was also reported [23]. The MCID for PRWHE was defined as a difference of 11.5 [24].

Complications were reported as follows:

- Sensory disturbance, including carpal tunnel syndrome and chronic regional pain syndrome;
- Flexor tendon rupture and irritation;
- Extensor tendon rupture and irritation;
- Hardware failure, e.g., osteosynthesis loosening;
- Infection: superficial (treated with antibiotics only) or deep (requiring a surgical intervention);
- Reoperation with hardware replacement;
- Reoperation with hardware removal (partial or total), which is not routinely performed in our country.

Pain was reported using the 0–10 numeric rating scale (NRS). Grip strength was measured using a calibrated dynamometer (EH101 CAMRY, by Camry^®^ Scales, South El Monte, CA, USA). The grip strength of both the left and right hands was estimated as the mean score of three repetition of each hand. Range of motion (ROM) was measured by one of the investigators using a goniometer.

Statistics

Basic demographic statistics were used to describe the study population. Two-way ANOVA was applied to determine if the observed effects were time-dependent, subject-dependent, or treatment-dependent. The significance level was set to *p* < 0.05.

The present trial was approved by the Danish Scientific Ethical Committee (ID: 1-10-72-420-17) and registered at Clinicaltrials.gov (ID: NCT03716661).

## 3. Results

**Figure 1:**
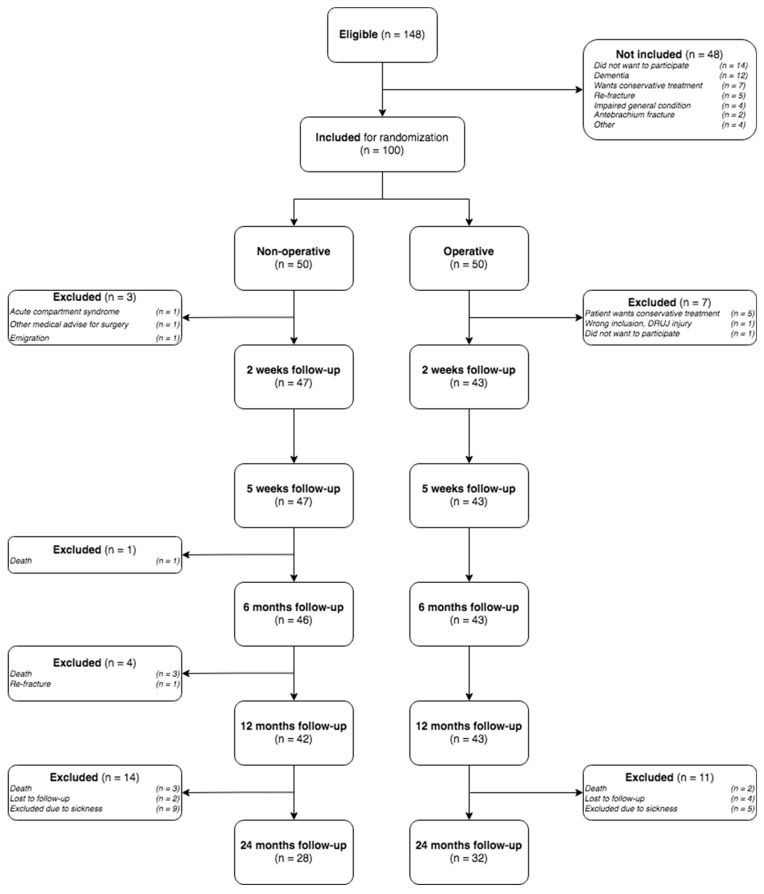
Consort flowchart.

After a median follow-up time of 3.0 (range 2.0–4.3) years, a total of 60 patients (28 non-operatively and 32 operatively treated DRFs) were available for data analysis at a minimum follow-up time of 2 years. Of the 85 patients available for the 1-year analysis of the published VOLCON RCT [14], 25 patients were lost to follow-up: a total of 5 patients were deceased, while 6 patients were lost to follow-up as they could not be contacted by telephone, and 14 patients did not want to participate in the study due to illness or a lack of time/interest (Figure 1).

Baseline demographics (age, gender, dominant hand fractured (yes/no), working status, and ASA class 1–6 (American Society of Anaesthesiologists Classification)) for the patients available for data analysis at a minimum 2-year follow-up and patients lost to follow-up between the 1- and 2-year follow-ups are given in Table 1, while the population at large from the VOLCON study can be found in the 1-year follow-up [14]. From years 1 to 2, the 25 patients lost to follow-up seemed to be slightly older but otherwise comparable to the included patients in terms of gender, occupational status, and ASA groups.

Explorative outcomes of the original RCT:

Radiological post-traumatic OA was assessed using pairs of radiographs at the 5-week follow-up and the latest follow-up at 2 years. The post-traumatic osteoarthritis grades are given in Table 2. Knirk and Jupiter describe these as follows: 0: none, 1: slight joint-space narrowing, 2: marked joint-space narrowing and osteophyte formation, and 3: bone-on-bone osteophyte formation and cyst formation [19]. According to two-way ANOVA analysis, time accounted for 25% (*p* < 0.001), the subject for 47% (*p* = 0.004), and treatment only for 0.3% (*p* = 0.57) of total variation.

**Figure 2:**
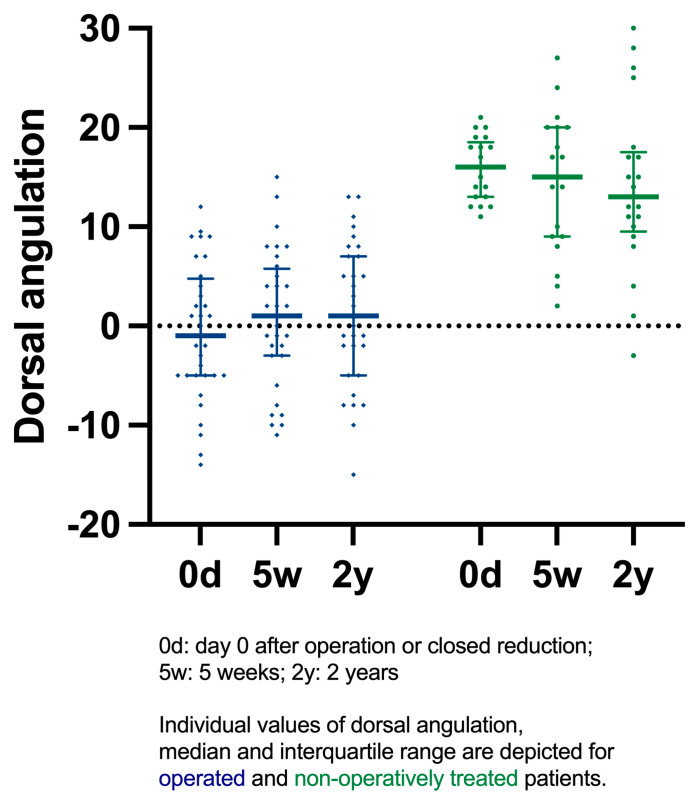
Dorsal angulation on day 0, 5 weeks, and 2 years.

The dorsal angulation was statistically significantly different between the treatment groups (*p* < 0.001), while time did not have a statistically significant impact when comparing immediate post-operative or closed reduction radiographs to radiographs after 5 weeks and 2 years (*p* = 0.978) (Figure 2). Notably, the AO/OTA classification was comparable with the AO type A/B/C distributed as follows: (19/4/9) in the operative group and (11/5/12) in the non-operative group.

The Quick-DASH from pre-injury, 2 weeks, 5 weeks, 6 months, 1 year, and 2 years was dependent on time (*p* < 0.001) and participant (*p* < 0.001) but not treatment (*p* = 0.56). If treatment had no effect overall, there was thus a 56% chance of randomly observing our results.

**Figure 3:**
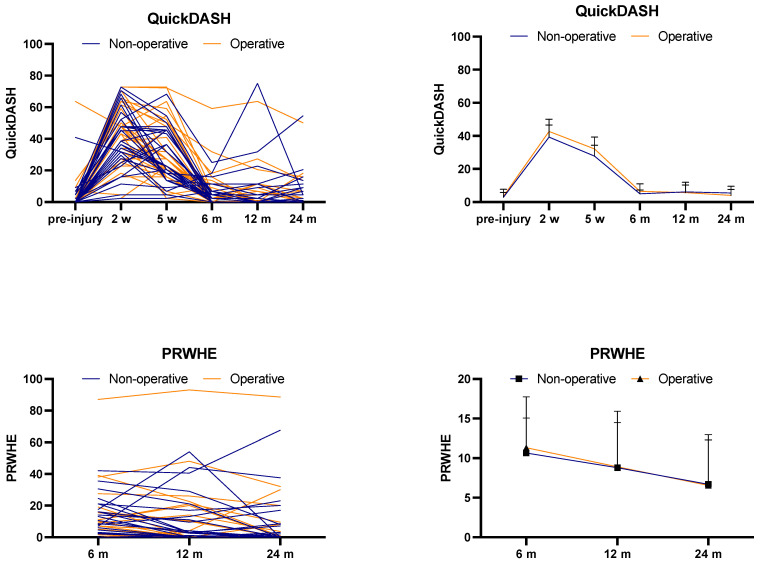
Mean Quick-DASH and PRWHE scores and mean scores at different time points.

Likewise, PRWHE scores at 6 months, 1 year, and 2 years were dependent on time (*p* = 0.01) and participant (*p* < 0.001) but not treatment (*p* = 0.96) (Figure 3). The mean Quick-DASH and PRWHE scores are presented in Table 3. Pain was also not treatment-dependent (*p* = 0.43), but it was time-dependent (Table 3). Likewise, ROM was not treatment-dependent, and the combined active movements, namely flexion–extension, ulnar–radial deviation, and supination–pronation, were all solely time-dependent. Combined flexion–extension ROM in the non-operative group vs. the operative group had a median of 105 (35–170) and 110 (45–170) degrees at the latest follow-up (*p* > 0.05). ROM in the other directions was also similar between groups; i.e., median combined pronation–supination scores of 180 (125–180) vs. 180 (135–180) and median combined deviation scores of 65 (35–75) vs. 55 (30–75) degrees (*p* > 0.05).

The mean grip strength of the fractured wrist was time-dependent (*p* < 0.001) but not treatment-dependent (*p* = 0.72). The mean grip strength of the fractured wrist was 15.4 kg, 17.3 kg, and 17.6 kg after 6 months, 1 year, and 2 years, respectively. The mean grip strength of the healthy side was not time-dependent (21.8 kg, 21.1 kg, and 20.5 kg; *p* = 0.07).

Complications between the 1- and 2-year follow-ups occurred predominantly in the operative group. For two non-operatively treated patients, one underwent surgery for carpal tunnel syndrome, while another one was diagnosed with De Quervain’s tenosynovitis and trigger finger, both of which required surgery. These complications were in addition to those observed within the first year, as reported in the VOLCON RCT [14]: two cases of superficial wounds at cast removal, two cases of surgically treated carpal tunnel syndromes, and three cases of nonspecific sensory disturbances within the first year.

In the operative group, two additional surgeries were performed between the 1- and 2-year follow-ups: one plate was removed due to extensor tendon irritation, and one patient underwent wrist arthrodesis. Furthermore, one proximal screw loosened but was not removed, and one patient had broken screws; however, the bone healed with increased dorsal angulation without requiring reoperation. Notably, the fluctuating sensory disturbances reported at 1 year were no longer present in the same patients at 2 years.

## 4. Discussion

In this minimum 2-year follow-up of an RCT investigating operative versus non-operative treatments of displaced DRFs in patients ≥65 years, we found no correlation between treatment choice and the development of post-traumatic OA. Furthermore, we found no difference in Quick-DASH, PRWHE, the pain score, ROM, or grip strength at the 2-year follow-up. As expected, there was significantly greater dorsal angulation in the non-operative group. Implant-related complications such as screw loosening and plate removal due to protruding screws causing tendon irritation were still observed between 1 and 2 years. Given the greater dorsal angulation in the non-operative group, it was surprising that one arthrodesis surgery was performed in a volar-plated DRF patient, while none of the non-operatively treated patients had bony surgery. However, a carpal tunnel release surgery for De Quervain’s tenosynovitis and trigger finger was performed in two non-operatively treated patients.

Our results regarding post-traumatic OA are comparable to Südow et al. [15] who also published an extension of an RCT comparing operative versus non-operative treatments of displaced DRFs in elderly patients. Similarly, they found no significant difference in OA at the 3-year follow-up. In contrast, another study found a significantly higher degree of post-traumatic OA in non-operatively treated DRFs with an indication for surgery. However, this study was retrospective, thus not randomized, and included a relatively small population of 50- to 70-year-old patients [25].

Several studies have found a correlation between malunion and articular step-off and the development of OA [19,26,27]. These studies, published in 1986, 1990, and 2011, included relatively young patients. However, Lutz et al. [27] concluded that there was no correlation between OA and the DASH score and grip strength and the pain score at the 9-year follow-up in a population with a mean age of 38 years. The relevance of these findings for an older population, such as in the present study, is unclear. We found a greater degree of dorsal angulation in the non-operative group compared to the operative group. According to these studies, this malunion could result in a higher degree of OA. However, we found similar OA rates in both groups. Additionally, functional outcomes were comparable between the two groups.

The etiology of post-traumatic OA in the wrist is believed to be multifactorial. A review on the topic suggests that ligament injuries and fractures are major contributing factors [28]. Injuries in other joints, such as the knee, have been associated with accelerated OA, particularly in the elderly [29,30]. Some have proposed intra-articular steroid injections to reduce the development of OA in DRFs post-operatively, but these failed to show any effect [31]. Interestingly, preexisting wrist or carpometacarpal OA did not affect postoperative functional outcomes (PRWHE or DASH) after DRFs in a retrospective case–control study including 61 patients [32].

We found no difference in Quick-DASH or PRWHE scores between the operative and non-operative groups at the 2-year follow-up, which aligns with findings from several meta-analyses and RCTs on operatively versus non-operatively treated DRFs [10,12,13,33,34,35,36]. However, these studies all had a maximum follow-up of 1 year. In contrast to our findings, some studies report better PROM scores in the operative group [37,38]. One of these meta-analyses found a lower Quick-DASH score of −5.22 (95% CI −8.87 to −1.57) in the operative group in the first year. However, in a subgroup analysis including elderly patients (>60 years), this difference diminished and was no longer significant [38]. Saving et al. [37] found a difference of 11.6 (8.3 vs. 19.9) in DASH scores favoring the operative group. Given that the MCID is defined as 16–20 points, the clinical relevance of this difference remains uncertain [20,21,22].

Three RCTs comparing operative versus non-operative treatments of DRFs with a minimum of two years of follow-up were identified [15,16,17]. Südow et al. concluded that the operative group had a small but statistically significant improvement in PRWHE scores (9-point difference) [15]. However, given that the MCID for PRWHE is 11.5, this difference is unlikely to be clinically relevant [24]. Additionally, they found no difference in DASH scores, which aligns with our findings. Likewise, Martinez-Mendez et al. [16] investigated operative versus non-operative treatments in patients > 60 years with intra-articular DRFs and found evidence of better PROM scores in the operative group at the 2-year follow-up. However, DASH scores remained below the MCID.

In another extension of a previously published RCT with a 2-year follow-up, Lawson et al. [17] found no difference in the pain score, PRWHE, the EuroQol−5 Domain (EQ5D), or complications supporting our results. Similar to another study [10], they found a higher degree of patient-reported treatment success in the operative group. The authors speculate whether this was due to earlier mobilization in the operative group or due to patients generally having a presumption that surgery is superior. Our study did not record patient expectations, but this may have been important, as patient expectations have been correlated to outcomes 12 months post-injury [39].

The Quick-DASH scores measured in the present study did not vary considerably from 6 to 24 months (Figure 3). This may be due to the inability of the PROMs to detect differences in patients rating themselves in the lower range of the instrument or due to patients adapting to a “new normal” and accepting decreased wrist function post-injury through adaptation and coping strategies. However, neither DASH nor PRWHE scores have been reported to show floor effects up to 9 months post-injury [40].

We found no difference in grip strength in the two groups. This finding is consistent with a comparable RCT with long-term follow-up [15] but contradicts most RCTs and meta-analyses with a one-year follow-up [13,33,35,37,38].

In this study, we had a small study population with a relatively high loss to follow-up, which is a considerable limitation. Of the 100 originally included patients, only 60 remained available for the final follow-up at a minimum of 2 years, resulting in a dropout rate of 40%. However, this is comparable to other RCTs [41,42,43]. Additionally, the sample size analysis for the original study was estimated to detect complication rates rather than post-traumatic OA [18]. While few studies have a follow-up period of at least 2 years, this may still be too short to detect post-traumatic OA development. Therefore, studies with larger sample sizes and longer follow-ups of up to 5–10 years are warranted. Finally, we used the classification of OA of the wrist proposed by Knirk and Jupiter [19], who included 43 patients with a mean age of 27 years. Our study population consisted of elderly patients > 65 years, and the applicability of this classification system to this age group is uncertain.

## 5. Conclusions

This study found no difference in post-traumatic OA or functional outcomes between operatively or non-operatively treated displaced DRFs in elderly patients > 65 years of age after 2 years of follow-up. This adds to the compiling evidence that the choice of treatment contributes little to the variability in functional and radiological outcomes after DRFs. However, longer follow-up studies are warranted to better assess the detection of post-traumatic OA as this study contributes to highlight short- to medium-term results.

## Figures and Tables

**Table 1:** Patient demographics for patients available for 2-year analysis and patients lost to follow-up from 1 to 2 years of follow-up.

	Available at Follow-Up at 2 Years		Lost to Follow-Up 1–2 Years	
	*n* = 60		*n* = 25	
	Non-Operative	Operative	Non-Operative	Operative
	*n* = 32	*n* = 28	*n* = 14	*n* = 11
Women [n/N (%)]	26/32 (81%)	22/28 (78%)	11/14 (79%)	8/11 (73%)
Fractured dominant side [n/N (%)]	13/32 (41%)	13/28 (46%)	8/14 (57%)	4/11 (36%)
Median age (range) [years]	73 (66–92)	72 (65–87)	78 (65–91)	78 (69–91)
Retired	31/32 (97%)	28/28 (100%)	14/14 (100%)	11/11 (100%)
ASA 1/ASA 2/ASA 3 [n]	10/19/3	11/16/1	2/9/3	3/7/1

ASA: American Society of Anaesthesiologists Classification.

**Table 2:** Post-traumatic osteoarthritis grade according to Knirk et al. [19].

	Non-Operative		Operative	
PA Grade	5 Weeks	2 Years	5 Weeks	2 Years
0	22	14	30	7
1	4	7	1	16
2	1	5	0	6
3	0	1	0	2

PA: Post-traumatic osteoarthritis.

**Table 3:** Quick-DASH, PRWHE, and pain scores 6 months, 1 year, and 2 years after the injury.

	Quick-DASH		PRWHE		Pain (NRS 0–10)	
	**Non-Operative**	**Operative**	**Non-Operative**	**Operative**	**Non-Operative**	**Operative**
6 months	**2.3** (0.0; 0.0–6.8; 25)	**2.3** (0.0; 0.0–6.8; 59)	**6.5** (0.0; 0.5–16; 42)	**7.0** (0.0; 0.0–15; 87)	**0** (0; 0–1; 5)	**0** (0; 0–1; 5)
1 year	**0.0** (0.0; 0.0–4.5; 75)	**0.0** (0.0; 0.0–6.2; 64)	**0.5** (0.0; 0.0–13; 54)	**0.0** (0.0; 0.0–10; 93)	**0** (0; 0–0; 5)	**0** (0; 0–0; 7)
2 years	**0.5** (0.0; 0.0–8.5; 55)	**0.0** (0.0; 0.0–2.3; 50)	**0.0** (0.0; 0.0–8; 68)	**0.0** (0.0; 0.0–3; 89)	**0** (0; 0–0; 5)	**0** (0; 0–0; 5)

Quick-DASH: Quick Disabilities of the Arm, Shoulder, and Hand. PRWHE: The Patient-Rated Wrist/Hand Evaluation. NRS: numerical rating scale.

## Data Availability

Data are unavailable due to privacy and ethical restrictions.

## Abbreviations

The following abbreviations are used in this manuscript:

DRF	Distal radius fracture
NCG	National clinical guideline
ED	Emergency department
ORIF	Open reduction internal fixation
PROMs	Patient-related outcome measures
OA	Osteoarthritis
Quick-DASH	Quick-disabilities of the arm, shoulder and hand
PRWHE	Patient-rated wrist/hand evaluation
MCID	Minimal clinical important difference
NRS	Numeric rating scale
ROM	Range of motion
ASA	American Society of Anaesthesiologists
EQ5D	EuroQol-5 Domain
RCT	Randomized controlled trial
